# GSK3α levels in Alzheimer´s disease mimicking models

**DOI:** 10.1002/alz.095568

**Published:** 2025-01-09

**Authors:** Beatriz Pinho, Margarida Vaz, Tânia Soares Martins, Odete da Cruz e Silva, Ana Gabriela Henriques

**Affiliations:** ^1^ Neurosciences and Signalling Group, Institute of Biomedicine (iBiMED), Department of Medical Sciences, University of Aveiro, Aveiro Portugal

## Abstract

**Background:**

Alzheimer’s disease (AD) is a progressive neurodegenerative disorder marked by the development of amyloid plaques and neurofibrillary tangles in the brain, which contribute to synaptic dysfunction and extensive neuronal loss. Protein phosphorylation is a key event in AD pathogenesis. Glycogen synthase kinase 3 (GSK3) is a serine/threonine kinase with two isoforms, GSK3α and GSK3β. While GSK3β link with AD is well stablished, the role of GSK3α in AD is not fully understood. Few studies suggest that GSK3α can be involved in tau phosphorylation, amyloid‐beta (Aβ) production, and neuronal cell death. Hence, the objective of this study was to test GSK3α levels and its potential contribution to AD.

**Method:**

Two AD‐mimicking models were employed, SH‐SY5Y treated with the Aβ peptide and SH‐SY5Y carrying the Swedish mutation of APP695. Cell lysates were subjected to Western Blot analysis to monitor total protein levels and phosphorylation of both GSK3 and its isoforms α and β.

**Result:**

Significant differences in the GSK3 total levels and in specific phosphorylation sites associated with protein activation were obtained for both isoforms in AD mimicking conditions.

**Conclusion:**

This research supports that GSK3α is altered in disease conditions and offers new insights of GSK3 involvement in AD pathology. This research was supported by iBiMED UIDB/04501/2020 and UIDP/04501/2020, FCT, the COMPETE program, QREN, and the European Union. M.V. is supported by the FCT through the individual PhD grant UI/BD/151354/2021.

